# End-of-life hospital referrals by out-of-hours general practitioners: a retrospective chart study

**DOI:** 10.1186/1471-2296-13-89

**Published:** 2012-08-22

**Authors:** Maria C De Korte-Verhoef, H Roeline W Pasman, Bart PM Schweitzer, Anneke L Francke, Bregje D Onwuteaka-Philipsen, Luc Deliens

**Affiliations:** 1 VU University medical center (VUmc), EMGO Institute for Health and Care Research, Department of Public and Occupational Health & Expertise Center Palliative Care VUmc, Amsterdam, the Netherlands; 2 VU University medical center, EMGO Institute for Health and Care Research, Department of General Practice, Amsterdam, the Netherlands; 3 NIVEL, Netherlands Institute for Health Services Research, Utrecht, the Netherlands; 4 Ghent University & Vrije Universiteit Brussel, End-of-life Care Research Group, Brussels, Belgium

**Keywords:** General Practice, Primary Care, Palliative Care, Out of Hours, Hospital Referral, Cancer, Symptoms

## Abstract

**Background:**

Many patients are transferred from home to hospital during the final phase of life and the majority die in hospital. The aim of the study is to explore hospital referrals of palliative care patients for whom an out-of-hours general practitioner was called.

**Methods:**

A retrospective descriptive chart study was conducted covering a one-year period (1/Nov/2005 to 1/Nov/2006) in all eight out-of-hours GP co-operatives in the Amsterdam region (Netherlands). All symptoms, sociodemographic and medical characteristics were recorded in 529 charts for palliative care patients. Multivariate logistic regression analysis was performed to identify the variables associated with hospital referrals at the end of life.

**Results:**

In all, 13% of all palliative care patients for whom an out-of-hours general practitioner was called were referred to hospital. Palliative care patients with cancer (OR 5,1), cardiovascular problems (OR 8,3), digestive problems (OR 2,5) and endocrine, metabolic and nutritional (EMN) problems (OR 2,5) had a significantly higher chance of being referred. Patients receiving professional nursing care (OR 0,2) and patients for whom their own general practitioner had transferred information to the out-of-hours cooperative (OR 0,4) had a significantly lower chance of hospital referral. The most frequent reasons for hospital referral, as noted by the out-of-hours general practitioner, were digestive (30%), EMN (19%) and respiratory (17%) problems.

**Conclusion:**

Whilst acknowledging that an out-of-hours hospital referral can be the most desirable option in some situations, this study provides suggestions for avoiding undesirable hospital referrals by out-of-hours general practitioners at the end of life. These include anticipating digestive, EMN, respiratory and cardiovascular symptoms in palliative care patients.

## Background

Many patients are transferred between care settings during the final phase of life [1-4]. In the final months of life, the most frequent trajectory of patients who die a non-sudden death is from home to hospital. The proportion following this trajectory ranges from 36 to 40% in the final three months of life in the Netherlands and Belgium to 68% in the final six months of life in Canada. The majority of patients who are transferred from home to hospital later die in hospital [5-7]. Two factors associated with hospital death are having spent at least one night in a hospital and the number of hospital admissions during the final year of life [8,9].

Hospital transfer, time spent in hospital at the end of life and hospital deaths are mentioned in the literature as poor end-of-life outcome indicators [10-12]. Although some end-of-life hospital transfers are necessary and could benefit the patient, most patients prefer to receive care and die at home, and most families evaluate staying at home as a desirable palliative pathway [13-15].

General practitioners (GPs) are key professionals in providing continuity of care at the end of life [16,17]. However, over the last two decades, the 24-hour availability of GPs has changed, with out-of-hours GP co-operatives at a greater distance and patients being more likely to receive care from a locum [18,19]; these changes could reduce the continuity of care [20,21]. Proper information transfer from the GP to the out-of-hours GP is an essential factor in optimizing continuity of care. In the Netherlands, 82% of GPs reported that they transferred information about terminally ill patients to out-of-hours GPs [22]. However, various studies of chart analyses of palliative care patients for whom an out-of-hours GP was called have shown that information from the patients’ own GP was available only for a minority [22-25].

In a study of patients referred to a palliative care programme, it was found that symptoms other than pain increase the number of transfers to in-patient care at the end of life [2]. However, no details were given about the type of symptoms. Other factors in addition to symptoms have also been found to increase the likelihood of hospital transfer at the end of life. Studies of general practices (not specifically limited to out-of-hours practices) show that age, gender, multiple morbidity, infections, respiratory problems, cardiovascular problems, a palliative treatment goal, GPs’ knowledge of patients’ wishes about the place of death and palliative treatment by the GP are all related to hospital transfers in the final three months of life [6,26].

The aim of our study was to explore hospital referrals of palliative care patients for whom an out-of-hours GP was called, in recognition of the fact that hospital transfers at the end of life can be undesirable for patients and their families and that the out-of-hours service might be a critical period for these referrals. The research questions were as follows. What is the incidence of hospital referrals of palliative care patients by out-of-hours general practices? What sociodemographic and medical characteristics and what symptoms presented by palliative care patients as noted by an out-of-hours GP are associated with end-of-life hospital referrals? What reasons for referrals from home to hospital are noted by the out-of-hours GP?

## Methods

### Design

A retrospective descriptive chart study was conducted looking at a one-year period (1/Nov/2005 to 1/Nov/2006) in all eight out-of-hours GP co-operatives in the Amsterdam region (Netherlands).

### Study population and setting

All 424 GPs in the Amsterdam region with local practices are also required to work shifts as locums for the eight out-of-hours GP cooperatives that serve the 800,000 inhabitants of Amsterdam. Patients who need help during the out-of-hours period can call a special number. Each patient call is noted in an electronic database known as Callmanager.

In the Netherlands, the GP is responsible for patients living at home and in homes for the elderly. Patients living at home have access to professional home care nurses, provided if there is a medical indication, while professional nurses are available 24 hours a day in homes for the elderly.

The Ethics Board of the VU University Medical Center, Amsterdam, was informed about the study, and they decided that the study did not require a formal ethical review.

### Patient calls

**Figure 1:**
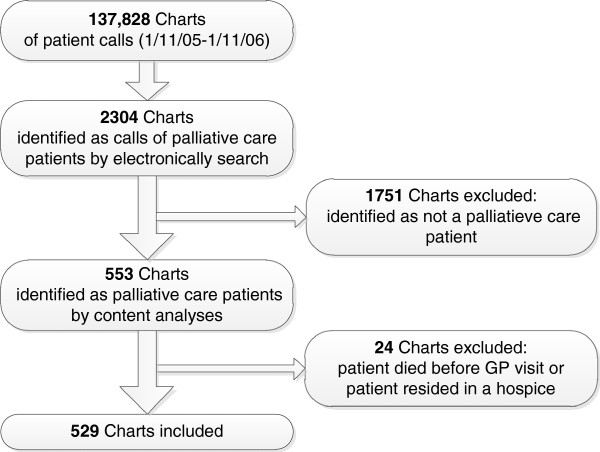
Flowchart: Selection of palliative-care patients in charts of 8 GP out-of-hours co-operatives in the Amsterdam region (Netherlands).

The total number of patient calls to the out-of-hours GP co-operatives during the one-year study period was 137,828. The records of all phone calls were screened electronically. Palliative care patients were identified by means of a search within the text for the words "palliative", "terminal", "cancer", "carcinoma", "inoperable", "opioid" and "fentanyl". The content of the 2304 records identified this way was subsequently examined by a GP with extensive experience in palliative care (BS). He included all contacts in which any mention was made of palliative care needs, palliative medication, remarks about terminal illness etc. This resulted in a list of calls for 553 different palliative care patients. The sensitivity of the search was checked by comparing the electronic search results with a manual search of data for all calls over a one-month period. This did not produce any new calls about palliative care patients and it was therefore decided that the manual search should be stopped [24]. Next, patients were excluded who died just before or during the locum’s visit or who were staying in a hospice. This left a total of 529 patients for whom an out-of-hours GP co-operative was called (Figure 1).

### Recording and analysis of the symptoms and care aspects

One of the authors (BS) analyzed the charts of palliative care patients for whom the out-of-hours GP co-operative was called (for a different paper about GP information transfer). For each patient we had one chart; for patients who had multiple contacts with the GP co-operative, only the final contact chart was included. The author recorded gender, age, type of residence (patient's home or home for the elderly), disease, a single main reason for the encounter, terminal status and hospital referral. Those results have been published elsewhere [24]. This paper is focusing on hospital referrals and this paper’s first author (MDK) therefore additionally recorded all the symptoms that were noted in the charts, all medical aspects and the reasons for hospital referral, and discussed this with the third author (BS). The symptoms were noted without interpreting possible mutual or causal relationships. We used the term hospital referral instead of hospital transfer because it is not clear from the charts whether the patient actually went to hospital.

Locums make a short report of the patient calls in a structured ‘SOEP’ registration system. The subjective reasons for the encounter are noted under S and the O contains the locum’s observations. The S and O categories provided the following variables: symptoms, family aspects, use of professional nursing care, patients’ wishes and medical aspects. The E category contains the evaluation of the situation from the locum’s perspective and also gives the reason for hospital referral. Finally, the plan - for instance the hospital referral - is noted in the P category. The symptoms were labelled in accordance with the main categories and subcategories of the International Classification of Primary Care (ICPC-2). If they were not described in the ICPC-2, they were classified according to the International Classification of Diseases (ICD10) [27,28]. Being terminally ill, the availability of family and receiving professional nursing care were counted as variables if the GP explicitly noted this in the chart. The symptoms presented in the tables are those that were noted for more than 5% of either of the two groups (patients with hospital referral and patients without hospital referral).

### Analysis

A *T*-test was used to compare the age and number of symptoms of referred patients with that of patients who had no hospital referral. A chi-squared test or Fisher’s exact test was used to assess the significance of differences in other sociodemographic and medical characteristics and in symptoms.

Multivariate logistic regression analysis was performed in order to identify the variables associated with hospital referral. First, univariate logistic regression was performed for each of the sociodemographic and medical characteristics (if noted for more than 5% of patients) and main categories of symptoms individually. All the significant variables (*P* < 0.05) were then entered in a stepwise backward multivariate logistic regression analysis (*P-value* for removal > 0.05). Because of the large overlap between place of residence (home for the elderly) and receiving professional nursing care, we only included ‘receiving professional nursing care’ in the multivariate analysis.

## Results

### Patient characteristics

In total, 13% of all palliative care patients for whom an out-of-hours GP co-operative was called were referred to hospital. Table 1 shows that 53% of all patients were male, the mean age was 73, 84% lived at home and 76% had cancer. Patients who were referred to hospital were significantly more likely to live at home (96% versus 82%), to have cancer (94% versus 73%) and to be receiving chemotherapy (12% versus 2%). In addition, patients for whom hospitalization was already planned within three days (10% versus 1%) and patients for whom a hospital specialist had already been contacted (9% versus 1%) were significantly more likely to be referred to hospital. Patients were significantly less likely to be referred to hospital if the patient was terminally ill according to the locum (75% versus 54%), if the patient was receiving professional nursing care (for the patients not living in a home for elderly: 30% versus 10%) or if information was available from the patients’ own GP (27% versus 9%).

**Table 1:** Sociodemographic and medical characteristics of palliative care patients as noted by an out-of-hours GP (N = 529)

		**Totaln = 529 %**	**Referraln = 68 %**	**No Referraln = 461 %**	***P*-value**
**Gender**					
	Male	53	47	54	0.283
**Age (years)**					
	Mean (SD)	73 (SD 14)	75 (SD 13)	73 (SD14)	0.297**
	≥ 75	50	52	50	0.795
**Residence**					
	Home	84	96	82	**0.006**
	Home for the elderly	16	4	18	
**Disease**					
	Cancer	76	94	73	**<0.001**
	Heart failure	4	4	4	0.743*
	COPD	3	0	3	0.234*
	Other diseases	18	1	20	**<0.001**
**Terminal status**					
	Terminally ill	73	54	75	**<0.001**
**Family**					
	Family available	59	62	58	0.594
	Family burden	7	9	7	0.455*
**Receiving professional nursing care**					
	Receiving professional nursing care	41	12	46	**<0.001**
**Patients wish**					
	Patients’ wish to stay at home	10	12	9	0.520
**Medical aspects**					
	Information transfer by patients’ GP	24	9	27	**0.002**
	Patient was receiving chemotherapy	4	12	2	0.001*
	Hospitalization was already planned within three days	2	10	1	<0.001*
	Patient or family had already contacted a hospital specialist	2	9	1	<0.001*

*Fisher Exact Test (2-sided).

** *T*-test.

### Symptoms

In total, 39 different symptoms were noted in the SOEP registration system. Pain (42%), dyspnoea (26%), agitation/confusion (19%), loss of appetite (19%), drowsiness (14%) and nausea/vomiting (16%) were the symptoms most commonly recorded (Table 2). Patients who were referred to hospital had significantly more symptoms (a mean of 3 versus a mean of 2) and were more likely to have digestive problems (53% versus 26%), endocrine, metabolic or nutrition (EMN) problems (46% versus 22%) or cardiovascular problems (13% versus 4%) than patients who were not referred. Looking in greater detail, the patients referred were more likely to have problems with nausea/vomiting (41% versus 12%), loss of appetite (35% versus 17%), dehydration (16% versus 3%), cachexia (12% versus 5%), pulmonary or deep venous thrombosis (7% versus 1%) and ileus (7% versus 2%).

**Table 2:** Symptoms as noted by an out-of-hours GP (N = 529) (the S and O in the SOEP registration)

**Main ICPC category**		**Totaln = 529 %**	**Referral n = 68 %**	**No Referraln = 461 %**	***P*-value**
	** Symptom**				
**General**		**48**	**52**	**48**	0.564
	Pain	42	46	41	0.495
	Fever ≥38	5	6	5	0.762*
	Other	3	4	3	0.446*
**Digestive**		**30**	**53**	**26**	**<0.001**
	Vomiting/ nausea	16	41	12	**<0.001**
	Swallowing problems	8	6	8	0.575
	Diarrhoea	3	6	3	0.137*
	Ileus	3	7	2	0.017*
	Ascites	2	6	2	0.056*
	Other	6	4	6	0.785*
**Respiratory**		**26**	**31**	**25**	0.335
	Dyspnoea	26	31	25	0.296
	Other	1	2	1	0.564*
**Endocrine, metabolic or nutritional**		**25**	**46**	**22**	**<0.001**
	Loss of appetite	19	35	17	**<0.001**
	Cachexia	6	12	5	0.042*
	Dehydration	4	16	3	<0.001*
	Other	2	3	2	0.625*
**Psychological**		**24**	**19**	**24**	0.444
	Agitation and confusion	19	15	19	0.385
	Other	8	4	9	0.249
**Neurological**		**16**	**9**	**17**	0.088
	Drowsiness	15	7	16	0.055
	Other	1	3	1	0.174*
**Urological**		**8**	**9**	**8**	0.812
**Cardiovascular**		**5**	**13**	**4**	**0.001**
	Pulmonary or deep venous embolism	2	7	1	0.005*
	Other	2	6	2	0.056*
**Skin problems**		**5**	**7**	**5**	0.387
**Number of Symptoms (mean, SD)**		**2.1 (SD 1.4)**	**3,0 (SD 1.6)**	**2,0 (SD 1.3)**	0.038**
	Patients with >2 symptoms	32	56	28	**<0.001**

*Fisher Exact Test (2-sided).

** *T*-test.

### Associations with hospital referral

Cancer, terminal illness, receiving nursing care, information transfer, digestive problems, EMN problems, cardiovascular problems and patients with more than two symptoms were significantly related to out-of-hours hospital referral in the univariate logistic regression analysis (*P* < 0.05) and were therefore included in the multivariate logistic regression analysis. Six variables remained significant (Table 3). Patients with cardiovascular problems (OR 8.3), cancer (OR 5.1), digestive problems (OR 2.5) and EMN problems (OR 2.5) were more likely to be referred to hospital. Patients who received professional nursing care (OR 0.2) and for whom their own GP had transferred information (OR 0.4) were less likely to be referred to hospital.

**Table 3:** Association with end-of-life hospital referral*

	**OR (95% CI)**
Cancer	5.1 (1.7-15.8)
Cardiovascular problems	8.3 (2.9-24,0)
Digestive problems	2.5 (1.4-4.6)
Endocrine, metabolic, and nutritional problems	2.5 (1.4-4.5)
Receiving professional nursing care	0.2 (0.1-0.5)
Information transfer by GP	0.4 (0.2-1.0)

*Stepwise backward multivariate logistic regression analysis.

The model demonstrated good calibration performance according to the Hosmer-Lemeshow goodness-of-fit test (*P* = 0.97) and a discriminatory ability of the area under the curve (AUC) of 0.83.

### Reasons for hospital referral

Twenty different symptoms and two other problems were noted as a reason for hospital referral from the perspective of the locum (Table 4). Digestive problems (31%), EMN problems (19%) and respiratory problems (18%) were the most common. At a more detailed level, vomiting (16%), dehydration (16%) and pneumonia/pleuritis (13%) were the reasons most often given for hospital referral. Two or three symptoms were mentioned for eleven patients and the family burden was mentioned for three patients.

**Table 4:** Reasons for hospital referral as noted by the out-of-hours GP (the E in the SOEP registration N = 68)

		**N**	**%**
**Digestive**		**21**	**30.9**
	Vomiting (incl. haematemesis)	11	16.2
	Ileus	4	5.9
	Ascites	3	4.4
	Rectal bleeding	2	2.9
	Peritonitis	1	1.5
**Endocrine, metabolic and nutritional**		**13**	**19.1**
	Dehydration	11	16.2
	Hyperglycaemia	2	2.9
**Respiratory**		**12**	**17.6**
	Pneumonia/pleuritis	9	13.2
	Dyspnoea	3	4.4
**Cardiovascular**		**8**	**11.8**
	Cardiac	4	5.9
	Deep venous thrombosis (feet)	3	4.4
	Pulmonary embolism	1	1.5
**General**		**7**	**10.3**
	Pain	5	7.4
	Weakness	1	1.5
	Fever	1	1.5
**Other symptoms**		**14**	**20.6**
	Epilepsy	6	8.8
	Anuria	4	5.9
	Confusion/delirium	3	4.4
	Fracture	1	1.5
**Other aspects**			
	Family burden or no family	6	8.8
	Diagnosis in the hospital	1	1.5

## Discussion

Examining 529 records of palliative care patients from out-of-hours GP co-operatives in the Amsterdam region, we found that 13% of palliative care patients were referred to a hospital. Palliative care patients with cancer, cardiovascular problems, digestive problems and endocrine, metabolic and nutritional (EMN) problems had a significantly higher chance of being referred after the out-of-hours consultation. Patients receiving professional nursing care and patients for whom their own GP had transferred information to the out-of-hours co-operative had a significantly lower chance of being referred after calling an out-of-hours GP. The reasons most commonly given for hospital referral were digestive problems, EMN problems and respiratory problems. The most common digestive problem was vomiting and the EMN problem noted most often was dehydration.

### Sociodemographic and medical characteristics

The multivariate analyses showed that palliative care patients had a lower chance of hospital referral in the out-of-hours period if they were receiving nursing care. Other studies have also found that patients receiving professional nursing care at the end of life are more likely to stay at home [29,30]. It is reasonable to assume that the nurse will in many cases know the patient’s situation and the care options at home better than a locum and will consequently be able to propose alternative ‘solutions’ to hospitalization. It therefore seems that nurses may considered as one of the ‘gatekeepers’ of out-of-hours hospitalization of palliative care patients. In addition to nursing involvement, it was also found that patients were less likely to be referred to hospital if their GP had transferred information to the locum. However, we also found that information from the patients’ own GP was only available for a minority of patients during the out-of-hours period. This lack of information transfer has also been noted in other studies looking at out-of-hours practices [22,23,25].

### Symptom*s*

Although 42% of the patients in this study were found to be in pain, this was often not the reason noted for out-of-hours hospital referrals. Symptoms such as digestive, EMN and respiratory problems were noted down more frequently as reason for referral. This suggests that out-of-hours GPs may be better at handling pain in palliative care patients than other symptoms.

As in our study, studies concerning out-of-hours care but not specifically focusing on palliative care patients also found that digestive problems are frequently recorded as a symptom [30,31]. We found digestive problems to be positively related to hospital referrals as well. About half of the referred patients in our study had digestive problems and 78% of these patients had problems with nausea and/or vomiting. Nausea/vomiting can have several causes, such as hypercalcaemia, obstipation, ascites, ileus or intracranial pressure [32]. The complexity of diagnosing or treating this might be a reason for hospital referral.

EMN problems were not found to be noted frequently in other studies [30,31], but in our study it was noted as a problem for 25% of the patients. EMN problems were also found to be significantly related to hospital referrals in our study. In the EMN category, 83% of the problems were caused by nutritional problems (loss of appetite, dehydration and cachexia). It is neither immediately apparent why an out-of-hours GP co-operative might be called for nutritional problems nor why out-of-hours GPs might consider this to be an acute reason for hospital referral.

### Strengths and limitations of the study

The strength of the study is the detailed information about sociodemographic and medical characteristics and symptoms of patients for whom an out-of-hours GP was called. A limitation of the study is that data was collected six years ago; however, there is no indication that GP services have changed substantially in the intervening years. Another limitation of the study is that problems were not measured systematically, for instance using a scoring list. Instead, the study used the reported problems as noted in the charts, which means that patients could have had additional problems. However, it is assumed that the problems noted were those causing the most distress. Furthermore, in our study it is possible that not all palliative care patients were detected by the electronic search strategy, although it should be noted that a manual search of a subset of the data did not uncover new patients. Additionally, it is not known whether the referred patients were indeed transferred to hospital after referral. Finally, we do not know if the hospital referrals found in our study could have been avoided or were undesirable.

## Conclusion

The results of this study provide detailed information about associations with hospital referrals, and the reasons for hospital referral outside standard hours. Whilst acknowledging that hospital referral can be the most desirable option in some situations, this study provides suggestions for avoiding undesirable hospital referrals at the end of life. Since it is not known how many of the hospital referrals were potentially avoidable and/or undesirable, further research should be done in this area. In order to anticipate potentially undesirable hospital transfers in out-of-hours periods, patients’ GPs could decide to provide information to out-of-hours GPs at an early stage, arrange for a nurse at home and be alert to digestive, nutritional and cardiovascular symptoms.

## Competing interest

The authors have declared that they have no competing interests.

## Authors’ contributions

MDK performed the draft and the statistical analyses of this paper together with HP. BS and MDK analyzed the charts of palliative care patients. HP and LD initiated the study and obtained the funding. HP, BOP, AF and LD supervised the project. All authors read, revised and approved the final manuscript.

## Pre-publication history

The pre-publication history for this paper can be accessed here:

http://www.biomedcentral.com/1471-2296/13/89/prepub
